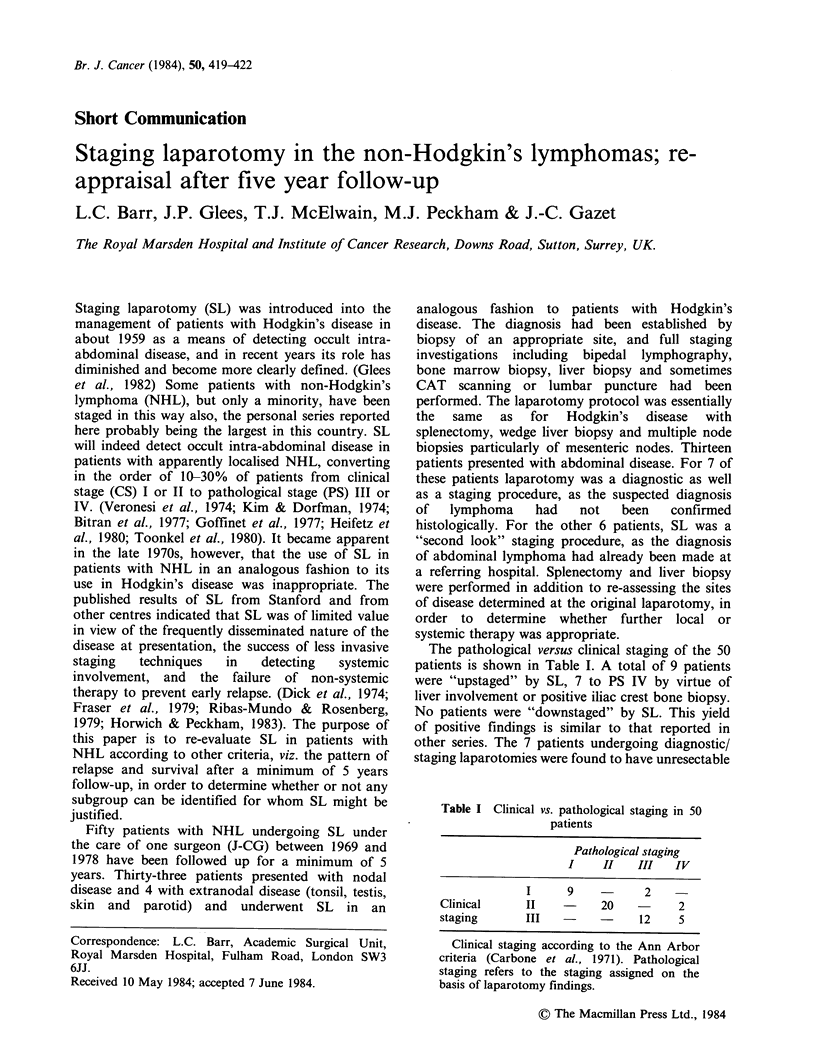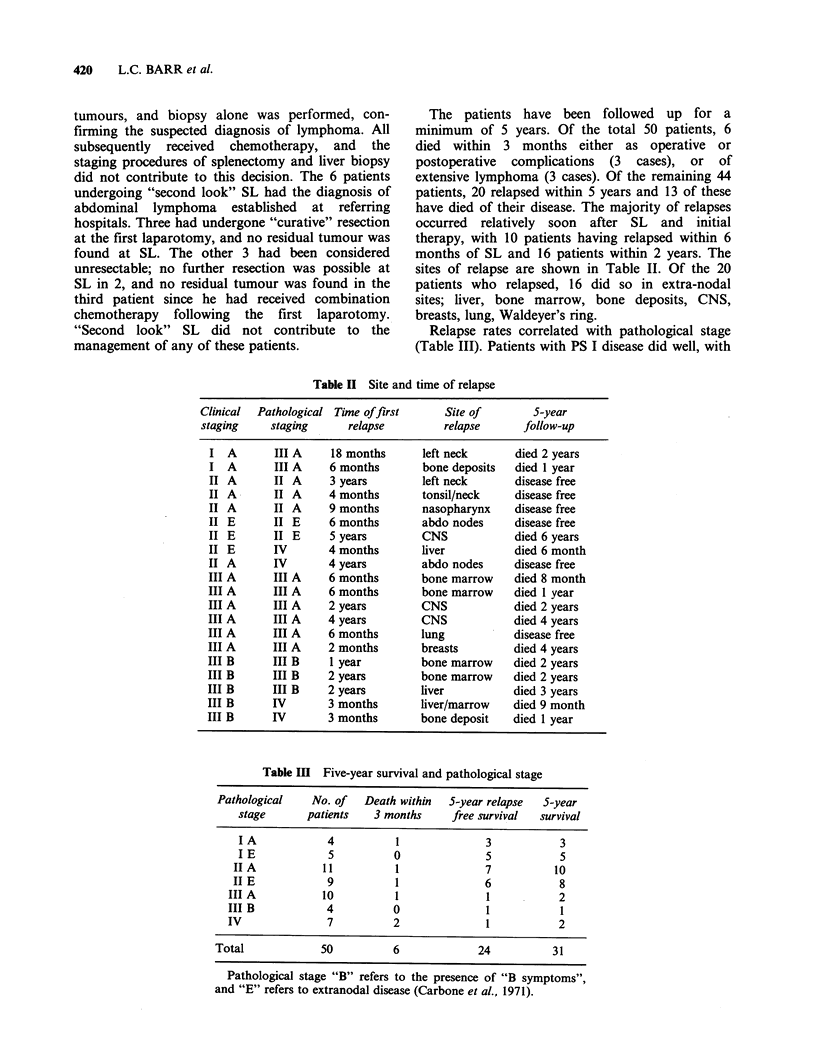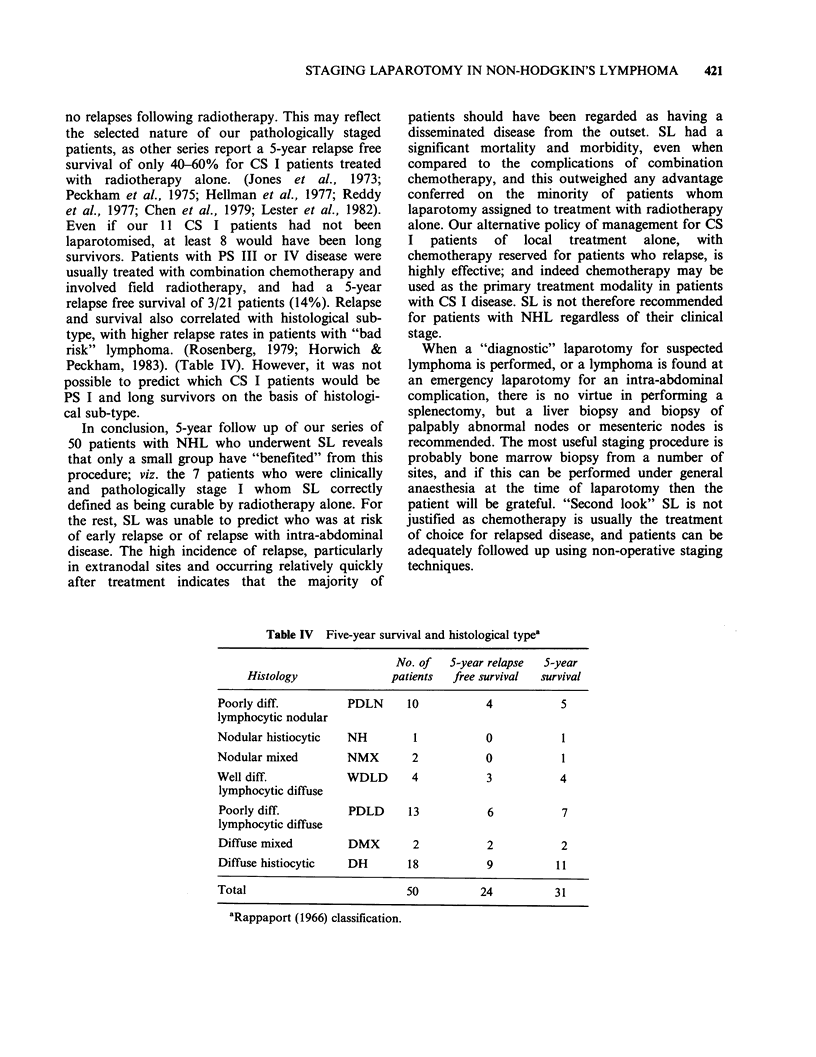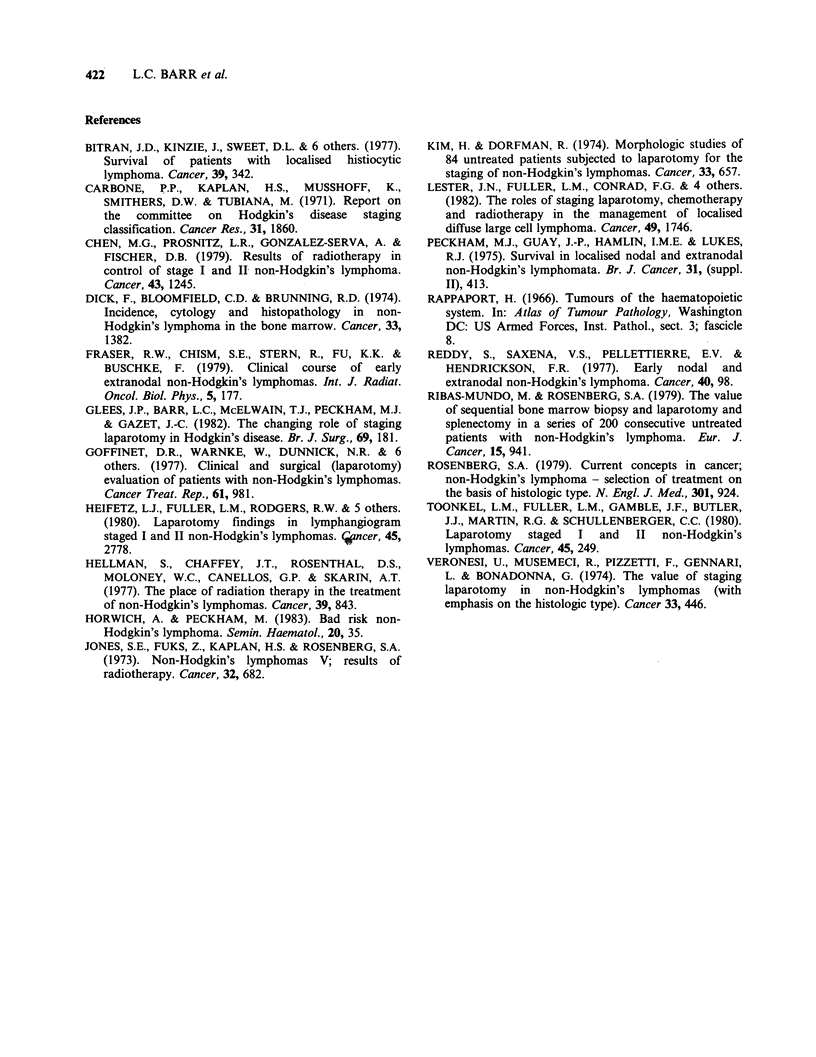# Staging laparotomy in the non-Hodgkin's lymphomas; reappraisal after five year follow-up.

**DOI:** 10.1038/bjc.1984.192

**Published:** 1984-09

**Authors:** L. C. Barr, J. P. Glees, T. J. McElwain, M. J. Peckham, J. C. Gazet


Br. J. Cancer (1984), 50, 419–422

**Short Communication**

### Staging laparotomy in the non-Hodgkin's lymphomas; re-appraisal after five year follow-up

L.C. Barr, J.P. Glees, T.J. McElwain, M.J. Peckham & J.-C. Gazet

*The Royal Marsden Hospital and Institute of Cancer Research, Downs Road, Sutton, Surrey, UK.*

Staging laparotomy (SL) was introduced into the management of patients with Hodgkin's disease in about 1959 as a means of detecting occult intra-abdominal disease, and in recent years its role has diminished and become more clearly defined. (Glees *et al.*, 1982) Some patients with non-Hodgkin's lymphoma (NHL), but only a minority, have been staged in this way also, the personal series reported here probably being the largest in this country. SL will indeed detect occult intra-abdominal disease in patients with apparently localised NHL, converting in the order of 10–30% of patients from clinical stage (CS) I or II to pathological stage (PS) III or IV. (Veronesi *et al.*, 1974; Kim & Dorfman, 1974; Bitran *et al.*, 1977; Goffinet *et al.*, 1977; Heifetz *et al.*, 1980; Toonkel *et al.*, 1980). It became apparent in the late 1970s, however, that the use of SL in patients with NHL in an analogous fashion to its use in Hodgkin's disease was inappropriate. The published results of SL from Stanford and from other centres indicated that SL was of limited value in view of the frequently disseminated nature of the disease at presentation, the success of less invasive staging techniques in detecting systemic involvement, and the failure of non-systemic therapy to prevent early relapse. (Dick *et al.*, 1974; Fraser *et al.*, 1979; Ribas-Mundo & Rosenberg, 1979; Horwich & Peckham, 1983). The purpose of this paper is to re-evaluate SL in patients with NHL according to other criteria, *viz.* the pattern of relapse and survival after a minimum of 5 years follow-up, in order to determine whether or not any subgroup can be identified for whom SL might be justified.

Fifty patients with NHL undergoing SL under the care of one surgeon (J-CG) between 1969 and 1978 have been followed up for a minimum of 5 years. Thirty-three patients presented with nodal disease and 4 with extranodal disease (tonsil, testis, skin and parotid) and underwent SL in an analogous fashion to patients with Hodgkin's disease. The diagnosis had been established by biopsy of an appropriate site, and full staging investigations including bipedal lymphography, bone marrow biopsy, liver biopsy and sometimes CAT scanning or lumbar puncture had been performed. The laparotomy protocol was essentially the same as for Hodgkin's disease with splenectomy, wedge liver biopsy and multiple node biopsies particularly of mesenteric nodes. Thirteen patients presented with abdominal disease. For 7 of these patients laparotomy was a diagnostic as well as a staging procedure, as the suspected diagnosis of lymphoma had not been confirmed histologically. For the other 6 patients, SL was a "second look" staging procedure, as the diagnosis of abdominal lymphoma had already been made at a referring hospital. Splenectomy and liver biopsy were performed in addition to re-assessing the sites of disease determined at the original laparotomy, in order to determine whether further local or systemic therapy was appropriate.

The pathological *versus* clinical staging of the 50 patients is shown in Table I. A total of 9 patients were "upstaged" by SL, 7 to PS IV by virtue of liver involvement or positive iliac crest bone biopsy. No patients were "downstaged" by SL. This yield of positive findings is similar to that reported in other series. The 7 patients undergoing diagnostic/staging laparotomies were found to have unresectable

**Table I** Clinical *vs.* pathological staging in 50 patients

| | | *Pathological staging* | | | |
|---|---|---|---|---|---|
| | | *I* | *II* | *III* | *IV* |
| | I | 9 | — | 2 | — |
| Clinical | II | — | 20 | — | 2 |
| staging | III | — | — | 12 | 5 |

Clinical staging according to the Ann Arbor criteria (Carbone *et al.*, 1971). Pathological staging refers to the staging assigned on the basis of laparotomy findings.

Correspondence: L.C. Barr, Academic Surgical Unit, Royal Marsden Hospital, Fulham Road, London SW3 6JJ.
Received 10 May 1984; accepted 7 June 1984.

© The Macmillan Press Ltd., 1984

tumours, and biopsy alone was performed, confirming the suspected diagnosis of lymphoma. All subsequently received chemotherapy, and the staging procedures of splenectomy and liver biopsy did not contribute to this decision. The 6 patients undergoing "second look" SL had the diagnosis of abdominal lymphoma established at referring hospitals. Three had undergone "curative" resection at the first laparotomy, and no residual tumour was found at SL. The other 3 had been considered unresectable; no further resection was possible at SL in 2, and no residual tumour was found in the third patient since he had received combination chemotherapy following the first laparotomy. "Second look" SL did not contribute to the management of any of these patients.

The patients have been followed up for a minimum of 5 years. Of the total 50 patients, 6 died within 3 months either as operative or postoperative complications (3 cases), or of extensive lymphoma (3 cases). Of the remaining 44 patients, 20 relapsed within 5 years and 13 of these have died of their disease. The majority of relapses occurred relatively soon after SL and initial therapy, with 10 patients having relapsed within 6 months of SL and 16 patients within 2 years. The sites of relapse are shown in Table II. Of the 20 patients who relapsed, 16 did so in extra-nodal sites; liver, bone marrow, bone deposits, CNS, breasts, lung, Waldeyer's ring.

Relapse rates correlated with pathological stage (Table III). Patients with PS I disease did well, with

**Table II** Site and time of relapse

| *Clinical staging* | *Pathological staging* | *Time of first relapse* | *Site of relapse* | *5-year follow-up* |
|---|---|---|---|---|
| I A | III A | 18 months | left neck | died 2 years |
| I A | III A | 6 months | bone deposits | died 1 year |
| II A | II A | 3 years | left neck | disease free |
| II A | II A | 4 months | tonsil/neck | disease free |
| II A | II A | 9 months | nasopharynx | disease free |
| II E | II E | 6 months | abdo nodes | disease free |
| II E | II E | 5 years | CNS | died 6 years |
| II E | IV | 4 months | liver | died 6 month |
| II A | IV | 4 years | abdo nodes | disease free |
| III A | III A | 6 months | bone marrow | died 8 month |
| III A | III A | 6 months | bone marrow | died 1 year |
| III A | III A | 2 years | CNS | died 2 years |
| III A | III A | 4 years | CNS | died 4 years |
| III A | III A | 6 months | lung | disease free |
| III A | III A | 2 months | breasts | died 4 years |
| III B | III B | 1 year | bone marrow | died 2 years |
| III B | III B | 2 years | bone marrow | died 2 years |
| III B | III B | 2 years | liver | died 3 years |
| III B | IV | 3 months | liver/marrow | died 9 month |
| III B | IV | 3 months | bone deposit | died 1 year |

**Table III** Five-year survival and pathological stage

| *Pathological stage* | *No. of patients* | *Death within 3 months* | *5-year relapse free survival* | *5-year survival* |
|---|---|---|---|---|
| I A | 4 | 1 | 3 | 3 |
| I E | 5 | 0 | 5 | 5 |
| II A | 11 | 1 | 7 | 10 |
| II E | 9 | 1 | 6 | 8 |
| III A | 10 | 1 | 1 | 2 |
| III B | 4 | 0 | 1 | 1 |
| IV | 7 | 2 | 1 | 2 |
| Total | 50 | 6 | 24 | 31 |

Pathological stage "B" refers to the presence of "B symptoms", and "E" refers to extranodal disease (Carbone *et al.*, 1971).

no relapses following radiotherapy. This may reflect the selected nature of our pathologically staged patients, as other series report a 5-year relapse free survival of only 40–60% for CS I patients treated with radiotherapy alone. (Jones *et al.*, 1973; Peckham *et al.*, 1975; Hellman *et al.*, 1977; Reddy *et al.*, 1977; Chen *et al.*, 1979; Lester *et al.*, 1982). Even if our 11 CS I patients had not been laparotomised, at least 8 would have been long survivors. Patients with PS III or IV disease were usually treated with combination chemotherapy and involved field radiotherapy, and had a 5-year relapse free survival of 3/21 patients (14%). Relapse and survival also correlated with histological sub-type, with higher relapse rates in patients with "bad risk" lymphoma. (Rosenberg, 1979; Horwich & Peckham, 1983). (Table IV). However, it was not possible to predict which CS I patients would be PS I and long survivors on the basis of histological sub-type.

In conclusion, 5-year follow up of our series of 50 patients with NHL who underwent SL reveals that only a small group have "benefited" from this procedure; *viz.* the 7 patients who were clinically and pathologically stage I whom SL correctly defined as being curable by radiotherapy alone. For the rest, SL was unable to predict who was at risk of early relapse or of relapse with intra-abdominal disease. The high incidence of relapse, particularly in extranodal sites and occurring relatively quickly after treatment indicates that the majority of patients should have been regarded as having a disseminated disease from the outset. SL had a significant mortality and morbidity, even when compared to the complications of combination chemotherapy, and this outweighed any advantage conferred on the minority of patients whom laparotomy assigned to treatment with radiotherapy alone. Our alternative policy of management for CS I patients of local treatment alone, with chemotherapy reserved for patients who relapse, is highly effective; and indeed chemotherapy may be used as the primary treatment modality in patients with CS I disease. SL is not therefore recommended for patients with NHL regardless of their clinical stage.

When a "diagnostic" laparotomy for suspected lymphoma is performed, or a lymphoma is found at an emergency laparotomy for an intra-abdominal complication, there is no virtue in performing a splenectomy, but a liver biopsy and biopsy of palpably abnormal nodes or mesenteric nodes is recommended. The most useful staging procedure is probably bone marrow biopsy from a number of sites, and if this can be performed under general anaesthesia at the time of laparotomy then the patient will be grateful. "Second look" SL is not justified as chemotherapy is usually the treatment of choice for relapsed disease, and patients can be adequately followed up using non-operative staging techniques.

**Table IV** Five-year survival and histological type[a]

| *Histology* | | *No. of patients* | *5-year relapse free survival* | *5-year survival* |
|---|---|---|---|---|
| Poorly diff. lymphocytic nodular | PDLN | 10 | 4 | 5 |
| Nodular histiocytic | NH | 1 | 0 | 1 |
| Nodular mixed | NMX | 2 | 0 | 1 |
| Well diff. lymphocytic diffuse | WDLD | 4 | 3 | 4 |
| Poorly diff. lymphocytic diffuse | PDLD | 13 | 6 | 7 |
| Diffuse mixed | DMX | 2 | 2 | 2 |
| Diffuse histiocytic | DH | 18 | 9 | 11 |
| Total | | 50 | 24 | 31 |

[a]Rappaport (1966) classification.

### References

BITRAN, J.D., KINZIE, J., SWEET, D.L. & 6 others. (1977). Survival of patients with localised histiocytic lymphoma. *Cancer*, **39**, 342.

CARBONE, P.P., KAPLAN, H.S., MUSSHOFF, K., SMITHERS, D.W. & TUBIANA, M. (1971). Report on the committee on Hodgkin's disease staging classification. *Cancer Res.*, **31**, 1860.

CHEN, M.G., PROSNITZ, L.R., GONZALEZ-SERVA, A. & FISCHER, D.B. (1979). Results of radiotherapy in control of stage I and II non-Hodgkin's lymphoma. *Cancer*, **43**, 1245.

DICK, F., BLOOMFIELD, C.D. & BRUNNING, R.D. (1974). Incidence, cytology and histopathology in non-Hodgkin's lymphoma in the bone marrow. *Cancer*, **33**, 1382.

FRASER, R.W., CHISM, S.E., STERN, R., FU, K.K. & BUSCHKE, F. (1979). Clinical course of early extranodal non-Hodgkin's lymphomas. *Int. J. Radiat. Oncol. Biol. Phys.*, **5**, 177.

GLEES, J.P., BARR, L.C., McELWAIN, T.J., PECKHAM, M.J. & GAZET, J.-C. (1982). The changing role of staging laparotomy in Hodgkin's disease. *Br. J. Surg.*, **69**, 181.

GOFFINET, D.R., WARNKE, W., DUNNICK, N.R. & 6 others. (1977). Clinical and surgical (laparotomy) evaluation of patients with non-Hodgkin's lymphomas. *Cancer Treat. Rep.*, **61**, 981.

HEIFETZ, L.J., FULLER, L.M., RODGERS, R.W. & 5 others. (1980). Laparotomy findings in lymphangiogram staged I and II non-Hodgkin's lymphomas. *Cancer*, **45**, 2778.

HELLMAN, S., CHAFFEY, J.T., ROSENTHAL, D.S., MOLONEY, W.C., CANELLOS, G.P. & SKARIN, A.T. (1977). The place of radiation therapy in the treatment of non-Hodgkin's lymphomas. *Cancer*, **39**, 843.

HORWICH, A. & PECKHAM, M. (1983). Bad risk non-Hodgkin's lymphoma. *Semin. Haematol.*, **20**, 35.

JONES, S.E., FUKS, Z., KAPLAN, H.S. & ROSENBERG, S.A. (1973). Non-Hodgkin's lymphomas V; results of radiotherapy. *Cancer*, **32**, 682.

KIM, H. & DORFMAN, R. (1974). Morphologic studies of 84 untreated patients subjected to laparotomy for the staging of non-Hodgkin's lymphomas. *Cancer*, **33**, 657.

LESTER, J.N., FULLER, L.M., CONRAD, F.G. & 4 others. (1982). The roles of staging laparotomy, chemotherapy and radiotherapy in the management of localised diffuse large cell lymphoma. *Cancer*, **49**, 1746.

PECKHAM, M.J., GUAY, J.-P., HAMLIN, I.M.E. & LUKES, R.J. (1975). Survival in localised nodal and extranodal non-Hodgkin's lymphomata. *Br. J. Cancer*, **31**, (suppl. II), 413.

RAPPAPORT, H. (1966). Tumours of the haematopoietic system. In: *Atlas of Tumour Pathology*, Washington DC: US Armed Forces, Inst. Pathol., sect. 3; fascicle 8.

REDDY, S., SAXENA, V.S., PELLETTIERRE, E.V. & HENDRICKSON, F.R. (1977). Early nodal and extranodal non-Hodgkin's lymphoma. *Cancer*, **40**, 98.

RIBAS-MUNDO, M. & ROSENBERG, S.A. (1979). The value of sequential bone marrow biopsy and laparotomy and splenectomy in a series of 200 consecutive untreated patients with non-Hodgkin's lymphoma. *Eur. J. Cancer*, **15**, 941.

ROSENBERG, S.A. (1979). Current concepts in cancer; non-Hodgkin's lymphoma – selection of treatment on the basis of histologic type. *N. Engl. J. Med.*, **301**, 924.

TOONKEL, L.M., FULLER, L.M., GAMBLE, J.F., BUTLER, J.J., MARTIN, R.G. & SCHULLENBERGER, C.C. (1980). Laparotomy staged I and II non-Hodgkin's lymphomas. *Cancer*, **45**, 249.

VERONESI, U., MUSEMECI, R., PIZZETTI, F., GENNARI, L. & BONADONNA, G. (1974). The value of staging laparotomy in non-Hodgkin's lymphomas (with emphasis on the histologic type). *Cancer* **33**, 446.